# Thrombospondin-1 Airway Expression and Thrombospondin-1 Gene Variants Are Associated with Bronchopulmonary Dysplasia in Extremely Low-Birth-Weight Infants: A Pilot Study

**DOI:** 10.3390/children12040424

**Published:** 2025-03-28

**Authors:** Parvathy Krishnan, Hannah Sampath, Van Trinh, Lance Parton

**Affiliations:** 1 Division of Newborn Medicine, The Regional Neonatal Intensive Care Unit, Maria Fareri Children’s Hospital at Westchester Medical Center, Valhalla, NY 10595, USA; lance.parton@wmchealth.org; 2 Tufts Medicine Pediatrics, Division of Newborn Medicine, Boston, MA 02111, USA; 3 Division of Neonatology, Children’s Hospital of Michigan, Detroit Medical Center, Detroit, MI 48201, USA; hannahsampath@gmail.com; 4 Division of Neonatology, Hutzel Women’s Hospital, Detroit Medical Center, Detroit, MI 48201, USA; 5 Department of Pediatrics, New York Medical College, Valhalla, NY 10595, USA; van7trinh@gmail.com

**Keywords:** BPD, TSP-1, gene variant in BPD, prematurity, neonate

## Abstract

Background: Thrombospondin-1 (TSP-1) is an extracellular glycoprotein that mediates the differentiation of pulmonary endothelial cells and specialized stem cells into alveolar epithelial lineage-specific cells during the repair phase after lung injury. Since bronchopulmonary dysplasia (BPD) involves the inhibition of lung development with altered lung structure and vasculature, differential expression of the THBS-1 gene may impact lung development and pulmonary endothelial cell repair and have an important role in BPD. Methods: This prospective single-center cohort study included ELBW infants with and without BPD. DNA from buccal swabs underwent RT-PCR with TaqMan probes, and TSP-1 protein was measured in tracheal aspirates. Statistical analyses used Chi-square tests, Fisher’s exact tests, Wilcoxon Rank Sum tests, and *t*-tests (*p* < 0.05). Results: ELBW infants with BPD had significantly lower gestational ages and birth weights compared to those without BPD [25 (24,26) and 27 (25,28) weeks; median (IQR); *p* = 0.008] and [712 (155) and 820 (153) grams; mean (SD); *p* = 0.002], respectively. There were significant differences in the haplotype distributions of THBS1 variants rs2664139/rs1478604 (*p* = 0.006) and THBS1 variants rs1478605/rs1478604 (*p* = 0.008) between no-BPD and BPD groups. There were also significant differences in airway TSP-1 protein levels between moderate and severe BPD patients [(*p* = 0.02) (no BPD: 527 (114–1755); moderate BPD: 312 (262–641); and severe BPD 211: (117–352) ng/dL; median (IQR)]. Conclusions: Although no individual variants differed, two THBS1 haplotypes and early TSP-1 airway expression varied by BPD severity, suggesting a role for TSP-1 in lung development and BPD pathogenesis in ELBW infants.

## 1. Introduction

Bronchopulmonary dysplasia (BPD) continues to pose a significant challenge in neonatal care, characterized by disrupted lung development and structural abnormalities affecting distal airspaces and vasculature. With advancements in perinatal care, encompassing the advent of surfactant therapy, prenatal corticosteroids, lung-protective mechanical ventilation techniques, vigilant management of patent ductus arteriosus, and enhanced nutritional support, among other interventions, the presentation and prognosis of BPD have been significantly altered. BPD now predominantly affects very immature lungs during the early stages of development even with lung-protective strategies and presents with impaired alveolarization manifesting as alveolar simplification and compromised vascular development [1,2]. There is little information on the etiology of this “new BPD”. The current understanding is that BPD in the post-surfactant era may originate from disruption of normal lung development. With premature birth, the normal sequence of lung development is disrupted, resulting in the histologic pattern of alveolar simplification (larger but fewer alveoli with decreased septation) and impaired or “dysmorphic” vascular growth [3]. Components of the extracellular matrix, which provide scaffolding and coordinate signaling within the matrisome, are also altered during lung development and injury resulting in BPD [4].

The molecular regulation of lung development and differentiation has been studied over the past 50 years [5]. Multiple signaling pathways have been identified as contributing to alveolarization and angiogenesis, such as elastin, platelet-derived growth factor, fibroblast growth factor, transforming growth factor, and others [6,7,8]. However, the interactions of these signal transduction pathways that regulate normal alveolar and pulmonary vascular development, and how these pathways are disrupted in premature infants with BPD, remain incompletely understood [9]. The subtle differences in the signaling pathways that regulate and coordinate lung alveolar and vascular development and differentiation may alter susceptibility to BPD.

Thrombospondin-1 (TSP-1) is a calcium-binding extracellular glycoprotein mapped to chromosome 15 that interacts with a multitude of proteins with an important role in the alveolar and vascular development of the lung [10,11,12,13]. TSP1 is highly expressed in lung endothelial cells and is upregulated during alveolar epithelial proliferation, differentiation, and repair. It interacts with the extracellular matrix, cytokines, cellular receptors, and growth factors and plays a key role of lung stem cell differentiation. TSP-1 binds multiple ligands and much of the biochemistry of TSP-1 has been ascertained in vitro [14]. However, the regulation of TSP-1 binding to many of these ligands and the net effect of these interactions in lung development have yet to be fully understood.

Animal studies have underscored the significance of TSP-1 in lung homeostasis, with TSP-1 deficiency exacerbating lung inflammation and impeding the resolution of injury [15]. Conversely, human studies are scarce, with even more limited exploration into the role of TSP-1 in BPD pathogenesis. Since TSP-1 interacts with signaling molecules including nitric oxide, as well as growth factors such as TGF-β, VEGF, and FGF2, which are key regulators in lung development, we sought to investigate whether there might be an association between early airway TSP-1 protein expression and BPD susceptibility in ELBW infants. In addition, we wanted to investigate whether selected variants of the *THSB1* gene—which codes for TSP-1—were also associated with BPD susceptibility. We chose candidate single-nucleotide polymorphisms (SNPs) of the *THSB1* gene that have been associated with lung function and disease in adults [16,17,18,19,20,21,22,23]. This is a pilot study investigating the relationship between genetic variants of the *THSB1* gene, early airway TSP-1 protein expression, and susceptibility to BPD in ELBW infants.

## 2. Materials and Methods

### 2.1. Study Population

This is a prospective, observational, longitudinal pilot study conducted at a single tertiary care center. The study population comprised infants with extremely low birth weight (ELBW), defined as a birth weight ≤1000 g. The infants were admitted to the regional neonatal intensive care unit at Maria Fareri Children’s Hospital at Westchester Medical Center in New York from time period ranging from 2012 to the present. Written informed consent was obtained from parents prior to enrollment. The enrolled infants were categorized into two groups: infants who developed bronchopulmonary dysplasia (BPD group) and those who did not (no-BPD group). The diagnosis of BPD was determined based on the requirement for supplemental oxygen at 36 weeks corrected gestational age, according to criteria established by the National Institutes of Health (NIH), categorizing BPD severity as moderate or severe.

Samples collected during the hospitalization period included buccal swabs and, from a subgroup of infants requiring mechanical ventilation, tracheal aspirates. Clinical data were prospectively extracted from electronic medical records. This study received approval from the Institutional Review Board (#8859) at New York Medical College and Westchester Medical Center [24].

### 2.2. DNA Extraction

Following informed consent, genomic DNA was collected non-invasively via buccal swabs obtained from infants during hospitalization. DNA extraction was performed using a commercial kit (QIAamp DNA extraction mini kit; Qiagen, Germantown, MD, USA) following the manufacturer’s instructions.

### 2.3. Genotyping

Genotyping of the candidate TSP-1 SNP was conducted using specific TaqMan probes and advanced genotyping master mix (Thermofisher, Pittsburgh, PA, USA). Real-time polymerase chain reaction was carried out on the Bio-Rad CFX96 (Bio-Rad, Philadelphia, PA, USA). The individual THBS-1 variants tested were rs2664139, rs1478604, rs1478605, rs2228262, and rs9879947. Initially, these five candidate SNPs within the THBS1 gene were selected for analysis. However, only three SNPs (rs2664139, rs1478604, and rs1478605) provided sufficient genotyping quality and call rates. Therefore, results and haplotype analyses reported here focus specifically on these three SNPs.

### 2.4. Tracheal Aspirate Analysis

Tracheal aspirates were collected within the first 10 days of life exclusively from infants requiring invasive mechanical ventilation. Samples were collected during routine suctioning procedures performed by experienced neonatal intensive care staff, using a strict sterile technique to minimize clinical disturbance and contamination.

The aspirate samples underwent immediate processing within one hour of collection. First, the samples were centrifuged at 500× *g* at 4 °C for 10 min to remove cellular debris. The resulting supernatant was further clarified by centrifugation at 10,000× *g* at 4 °C for another 10 min. The cell-free supernatants were then aliquoted and stored at −80 °C until batch analysis.

Quantification of TSP-1 levels in the tracheal aspirate supernatants was conducted using sandwich enzyme-linked immunosorbent assay (ELISA), employing the Human Thrombospondin-1 Quantikine ELISA Kit (Catalog # DTSP10; R&D Systems, Inc., Minneapolis, MN, USA). While standardization methods (e.g., dilutional markers like blood urea nitrogen) remain debated, we maintained consistent collection and processing protocols to minimize variability.

#### Statistical Analysis

Demographic data of the continuous variables were analyzed by Student’s *t*-tests or Mann–Whitney tests, and categorical variables were analyzed by Chi-square or Fisher’s exact tests. Gene polymorphism correlations between the two cohorts were examined using the Chi-square test. Allele frequencies were evaluated by Z tests. Comparative analysis of early airway protein expression was performed using the Mann–Whitney Wilcoxon rank sum test. A *p*-value < 0.05 was considered significant with Bonferroni correction for demographic variables. Statistical analysis was conducted using Sigma Plot version 13 and SPSS version 26.

## 3. Results

### 3.1. Demographics

Demographic and maternal characteristics of the infants who developed BPD (*n* = 67) and who did not develop BPD (no BPD; *n* = 30) are shown in Table 1. Birth weights and gestational ages were significantly lower in the BPD group compared to the no-BPD group, as expected [712 (155) and 820 (153) g, mean (SD); 25 (24,26) and 27 (25,28) weeks, median (IQR), respectively]. Incidences of patent ductus arteriosus (PDA), necrotizing enterocolitis (NEC), and IVH (grade 3 or above) were not different between the two groups.

### 3.2. TSP-1 Genotyping

Genotype distributions, minor allele frequencies, and the presence of any minor alleles of these variants were not significantly different between the BPD and no-BPD groups on the 3 SNPs (rs2664139, rs1478604, and rs1478605) (Table 2). There were differences in certain haplotype distributions for those with BPD compared to those with no BPD when the rs1478604 variant was combined with either rs2664139 (*p* = 0.006) or rs1478605 (*p* = 0.008) (Table 3). Pairwise haplotype analysis was conducted due to the limited sample size of this pilot cohort. After adjusting for birth weight, male sex, NEC, IVH grade 3 or above, and PDA treatment, the adjusted *p*-values were 0.02 and 0.04, respectively.

### 3.3. Airway TSP-1 Protein Expression

**Figure 1:**
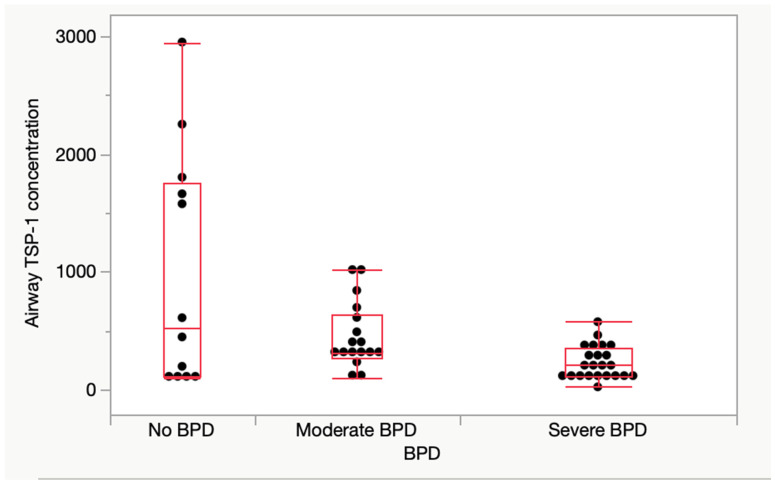
Early airway TSP-1 protein expression in the study population. Early airway TSP-1 protein levels in the 52 infants with no, moderate, or severe BPD. The median TSP-1 expression in the subset was 280 (139–460). TSP-1 protein level was significantly different between the groups [no BPD: 527 (114–1755), moderate BPD 312 (262–641), and severe BPD 211 (117–352) ng/dL; median (IQR), *p*-value = 0.02]. There was a significant difference in the early airway protein expression between the severe BPD and moderate BPD groups (*p* = 0.01). Abbreviations: TSP-1—thrombospondin-1 protein, BPD—bronchopulmonary dysplasia, IQR—interquartile range.

Early airway TSP-1 protein levels were assessed in 52 infants, which formed a subset of our total sample. The median TSP-1 expression in the subset was 280 ng/dL (139–460). Early airway TSP-1 protein levels were significantly different between the group with severe BPD and that with moderate BPD. There was a progressive decreasing trend in TSP-1 protein levels with increasing BPD severity [no BPD: 527 (114–1755), moderate BPD: 312 (262–641), and severe BPD 211: (117–352) ng/dL; median (IQR)] (Figure 1). All aspirates were collected under standardized conditions within the first 10 days of life to ensure comparability of measurements.

## 4. Discussion

This study investigated the associations of *THSB1* gene variants and early airway TSP-1 protein expression in ELBW infants with and without BPD. Although our data did not show any differences in the distribution of the individual *THSB1* gene variants rs2664139, rs1478604 and rs1478605 in infants with BPD as compared to infants without BPD, there was a difference in the haplotype distribution for those with BPD compared to those with no BPD when the rs1478604 variant combined with either rs2664139 or rs1478605. We also found significant differences in early airway TSP-1 expression between the group with severe BPD and that with moderate BPD, with a decline in TSP-1 airway expression associated with increasing BPD severity.

The variant rs1478604 is found in the 5′-UTR with a minor allele frequency of 0.42. Due to its location, it has the potential to influence transcription. Indeed, a decrease in translation has been seen in an ocular study [25]. This SNP has been associated with inflammation and cardiopulmonary functions. The variant rs2664139 is an Upstream Transcript Variant with a minor allele frequency of 0.43 T > c, and rs1478605 is a 5 Prime UTR Variant with a minor allele frequency of 0.42 G > a.

Gain and loss of function experiments have revealed the crosstalk between TSP-1 and BMP4 pathways in the inducing alveolar differentiation [26]. Gene expression during increased lung growth induced by fetal lung expansion in animal models identified TSP-1 as one of the most upregulated genes by 200–300%, implicating a role for TSP-1 in lung growth and development [27,28]. TSP-1 expressed in alveolar epithelial cells and fibroblasts increases up to 3.5-fold during lung development in fetal life, underlining its mechanotransduction potential [15]. TSP-1 has been shown to direct the differentiation of bronchioalveolar stem cells (BASCs) into type II alveolar epithelial (ATII) cells, which synthesize and release surfactants and are considered to be the main progenitor cell type [26]. TSP1 has been shown to induce BMP4 and activate calcineurin signaling and alveolar differentiation. TSP-1 binds to the integrins and mechanoreceptive ion channels, which transmit extracellular signals and induce intracellular changes. In contrast, our early airway TSP-1 expression of decreasing TSP-1 levels with increasing severity of BPD implicates an important association of TSP-1 expression with lung development and injury. This is consistent with a proposed role for TSP-1 and BMP4 interactions in early lung development and injury.

**Figure 2:**
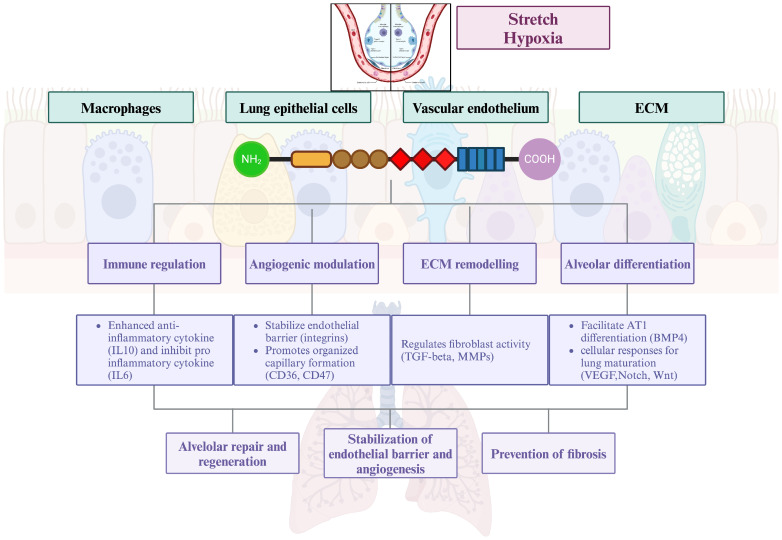
Proposed mechanism by which TSP-1 signaling interactions affect lung development. Abbreviations: TSP-1—thrombospondin-1 protein, ECM—extracellular matrix, IL—interleukin, TGF—tumor growth factor, MMP—matrix metalloproteinase, AT—alveolar epithelial type. Created with BioRender.com, accessed on 11 September 2024.

The role of TSP-1 as a key regulator of bronchoalveolar stem cell differentiation also has important implications in the context of lung regeneration and repair after injury. In the absence of TSP-1, BASCs are more likely to differentiate into bronchiolar epithelial cells and result in defective alveolar type II epithelial regeneration. TSP-1 inhibits neutrophil serine proteolytic functions such as neutrophil elastase (NE) and cathepsin G (CG) and facilitates macrophage IL-10 production following contact recognition of apoptotic neutrophils [29]. TSP-1 knockout mice develop spontaneous noninfectious pneumonia and lung inflammation, with findings of exaggerated neutrophilic inflammation and increased lung microvascular permeability during the acute phase of injury, as well as a defective resolution [30,31,32]. TSP-1 deficiency leads to elevated expression of collagen as well as connective tissue growth factor (CTGF), a downstream gene product of TGF-b [33,34,35,36,37]. Overall, TSP-1 works in a paracrine fashion to limit the extent and/or duration of inflammation during the host response to lung injury [38,39], which may play an important role in alveolar epithelial cell regeneration following respiratory distress syndrome and may be a critical target for the diagnosis and therapeutics of BPD (Figure 2). Given the limitations inherent in the clinical definitions of BPD, longitudinal serial measurements of biomarkers like TSP-1 could offer valuable predictive insights. Monitoring TSP-1 expression over critical periods in early postnatal lung development may help validate severity criteria, predict long-term pulmonary morbidity, and identify infants who might benefit from targeted therapeutic strategies.

To the best of our knowledge, this is the first study evaluating the role of genetic variants of TSP-1 and early airway protein expression of TSP-1 protein in ELBW infants in the context of BPD. We speculate that, given the key role of TSP-1 in alveolar differentiation and lung repair, the observed difference in the haplotype distribution of *THSB1* gene variants between the no-BPD and BPD groups may indicate the difference in downstream expression for the haplotype combination that may preserve the lung development and repair processes in preterm infants.

Our study is best interpreted in the context of its limitations. Firstly, this was a longitudinal prospective cohort giving rise to variation in clinical practice that may affect the phenotype. Secondly, we used the NICHD definition of BPD based on oxygen supplementation for simplicity; however, the clinical nature of this definition and its uncertain association with long-term outcomes may limit the generalizability of our findings. Thirdly, sample size variations due to SNP call rate differences and lack of epigenetic analysis are notable limitations. Additionally, variability in tracheal aspirate collection timing and response to surfactants may influence TSP-1 levels. While standardization of protein concentration using dilutional markers such as BUN has been debated, we ensured uniform collection procedures throughout the study period to minimize variability. Moreover, protein expression data were not linked directly to genotypes or clinical outcomes such as in-hospital mortality, ICU length of stay, or invasive mechanical ventilation requirements, due to the limited sample size. Further studies should explore these potential associations. Future studies incorporating multi-center data and additional inflammatory markers alongside clinical outcomes are needed to evaluate the role of *THBS1* expression in BPD.

Further studies should explore the impact of epigenetic modifications on *THBS1* expression and BPD risk. Larger, multi-center cohorts are needed to validate these findings and assess the interaction between genetic variants and environmental exposures. Standardizing the timing of tracheal aspirate collection and correlating TSP-1 levels with genotype data could provide deeper insights into its mechanistic role in lung development. Additionally, functional studies investigating TSP-1’s role in endothelial and epithelial lung repair pathways may elucidate potential therapeutic targets for BPD prevention and management.

## 5. Conclusions

This pilot study investigated the association of *THSB-1* gene polymorphism and early airway protein expression in infants with and without BPD. Our data show that the individual *THSB-1* gene variants rs2664139, rs1478604 and rs1478605 are not associated with susceptibility to BPD. We found a difference in the haplotype distribution for those with BPD compared to those with no BPD when variant rs1478604 was combined with one of the other variants. Early airway TSP-1 expression was significantly different among the non-BPD, moderate BPD, and severe BPD groups, with the most affected infants having the lowest airway TSP-1 expression. This implicates TSP-1 as a regulator of alveolar differentiation and repair and suggests that variants of *THSB-1* known to decrease expression may abrogate lung development and the response to injury by locally decreasing TSP-1 levels. We speculate that this decreased TSP-1 expression in ELBW infants with BPD may be susceptible to immunomodulatory drugs (IMiDs) [40] and synthetic TSP-1 mimetics [41], thereby offering additional therapeutic opportunities for BPD.

## Figures and Tables

**Table 1:** Demographic and clinical characteristics of the study population.

		No BPD (n = 30)	BPD (n = 67)	*p*-Value	Corrected *p*-Value
GA, weeks., median (IQR)		27 (25,28)	25 (24,26)	**0.008**	0.16
BW, g, mean (SD)		820 (153)	712 (155)	**0.002**	**0.04**
SGA, n (%)		5 (17)	12 (18)	0.91	1
Male gender, n (%)		12 (40)	38 (57)	0.11	1
Race, n (%)	**NH white**	8 (27)	22(33)	0.52	1
	**NH black**	11 (37)	16(24)		
	**Hispanic**	7 (23)	17 (25)		
	**Other**	4 (13)	12 (18)		
Maternal age, mean (SD)		28 (7)	28 (7)	0.97	1
C section delivery, n (%)		22 (75)	44 (71)	0.62	1
Multiple birth, n (%)		11 (37)	16 (25)	0.23	1
Inborn, n (%)		22 (76)	48 (76)	0.97	1
Antenatal steroids, n (%)		26 (90)	57 (86)	0.65	1
PROM, n (%)		6 (22)	14 (23)	0.97	1
Chorioamnionitis, n (%)		5 (17)	7 (11)	0.37	1
Preeclampsia, n (%)		8 (27)	18 (27)	0.89	1
Gestational diabetes, n (%)		4 (13)	11(16)	0.69	1
5 min APGAR < 7, n (%)		11 (37)	33 (50)	0.22	1
Surfactant, n (%)		26 (87)	56 (83)	0.92	1
Sepsis, n (%)		7 (23)	16 (24)	0.98	1
Any PDA treatment, n (%)		19 (63)	52 (78)	0.11	1
NEC, n (%)		3 (10)	15 (22)	0.18	1
IVH grade 3 or above, n (%)		1 (3)	10 (15)	0.08	1

The study population was divided into 2 groups: those who developed BPD (BPD, *n* = 67) and those who did not (no BPD, *n* = 30). The table shows maternal and infant characteristics of the two groups. *p*-Values were adjusted using the Bonferroni correction for multiple comparisons. The correction was applied by multiplying the raw *p*-values by the number of comparisons, and values greater than 1 were capped at 1.0. Abbreviations: BPD—bronchopulmonary dysplasia, GA—gestational age, BW—birth weight, SGA—small for gestational age, NH—non-Hispanic, PROM—prolonged rupture of membranes, PDA—patent ductus arteriosus, NEC—necrotizing enterocolitis, IVH—intraventricular hemorrhage, SD—standard deviation, IQR—interquartile range.

**Table 2:** Genotype distribution of the THBS1 gene variants in the study population.

rs2664139	BPD N = 62	No BPD N = 27	*p*-Value
TT, n (%)	45 (73%)	15 (56%)	0.26
Tc, n (%)	8 (13%)	5 (18%)	
cc, n (%)	9 (14%)	7 (26%)	
Any minor alleles (c), n (%)	17 (27%)	12 (44%)	0.11
MAF	0.35	0.21	0.13
**rs1478605**	**BPD** **N = 58**	**No BPD** **N = 24**	
GG, n (%)	26 (45%)	9 (35%)	0.26
Ga, n (%)	18 (31%)	5 (22%)	
aa, n (%)	14 (24%)	10 (43%)	
Any minor alleles (a), n (%)	32 (57%)	15 (65%)	0.54
MAF	0.39	0.52	0.28
**rs1478604**	**BPD** **N = 51**	**No BPD** **N = 15**	
TT, n (%)	22 (43%)	7 (47%)	0.27
Tc, n (%)	20 (39%)	3 (20%)	
cc, n (%)	9 (18%)	5 (33%)	
Any minor alleles (c), n (%)	29 (57%)	8 (53%)	0.80
MAF	0.37	0.43	0.67

The THBS1 genotype distributions of rs2664139 [MAF = 0.43 T > c (1000 genome data)], rs1478605 [MAF = 0.43 G > a (1000 genome data)], and rs1478604 [MAF = 0.42 T > c (1000 genome data)] were compared between the BPD and no-BPD groups. The presence of any minor alleles and the MAF were also compared between these two groups. Abbreviations: *THBS1*—thrombospondin-1 gene, BPD—bronchopulmonary dysplasia, MAF—minor allele frequency.

**Table 3:** Haplotype distribution.

Genotype Distribution (rs2664139 and rs1478604)	BPD	No BPD	Unadjusted *p*	Adjusted *p*
No minor alleles (%)	44%	34%	**0.006**	**0.02**
1 or more minor alleles (%)	44%	0		
Homozygous minor alleles (%)	12%	67%		
Genotype distribution (rs1478605 and rs1478604)	**BPD**	**No BPD**		
No minor alleles (%)	52%	44%	**0.008**	**0.04**
1 or more minor alleles (%)	36%	0		
Homozygous minor alleles (%)	12%	56%		

Haplotype distributions of THBS1 variants rs2664139/rs1478604 and rs1478605/rs1478604. Distributions were compared between BPD and no-BPD groups and were found to be significantly different (unadjusted *p* < 0.05). *p*-Value was adjusted for birth weight, male sex, NEC, IVH grade 3 or above, and PDA treatment. Abbreviations: *THBS1*—thrombospondin-1 gene, BPD—bronchopulmonary dysplasia.

## Data Availability

New York Medical College and Westchester Medical Center have archived the dataset analyzed.

## Abbreviations

The following abbreviations are used in this manuscript:

BPD	Bronchopulmonary Dysplasia
ELBW	Extremely Low Birth Weight
TSP-1	Thrombospondin-1
SNP	Single-Nucleotide Polymorphism
TGF-β	Transforming Growth Factor Beta
VEGF	Vascular Endothelial Growth Factor
BASCs	Bronchioalveolar Stem Cells
ATII	Alveolar Type II
LPS	Lipopolysaccharide
NE	Neutrophil Elastase
CG	Cathepsin G
CTGF	Connective Tissue Growth Factor
IL-10	Interleukin-10
LSKL	Leucine–Serine–Lysine–Leucine
LAP	Latency-Associated Peptide
RS	Reference SNP Cluster ID
